# Spectroscopic and Microscopic Analysis of Apple Pectins

**DOI:** 10.3390/molecules30071633

**Published:** 2025-04-06

**Authors:** Agata Serrafi, Agnieszka Wikiera, Konrad Cyprych, Magdalena Malik

**Affiliations:** 1 Department of Immunochemistry and Chemistry, Medical University of Wroclaw, ul. M. Sklodowskiej-Curie 48/50, 50-369 Wroclaw, Poland; agata.serrafi@umw.edu.pl; 2 Faculty of Health Sciences, Jagiellonian University Medical College, 31-008 Krakow, Poland; agnieszka.wikiera@uj.edu.pl; 3 Faculty of Chemistry, Wroclaw University of Science and Technology, Wyb. Wyspianskiego 27, 50-370 Wroclaw, Poland; konrad.cyprych@pwr.edu.pl

**Keywords:** pectins, structural properties, morphological analysis, mass spectroscopy, FT-IR, NMR

## Abstract

Apple pomace pectins, extracted using various methods (water, cellulase, arabinase, and arabinase with mannanase), and commercial apple pectin were studied, analyzing their morphology and chemical structure. The microscopic analysis revealed morphological differences, with a log-normal particle size distribution observed in most samples, except for those extracted with water. Cellulase-extracted pectin exhibited the most spherical morphology, while enzymatically extracted pectins displayed uneven surfaces. The FT-IR analysis indicated structural changes, shifts in O-H bands, and the degree of methoxylation (DM) ranged from 30.25% to 58%, with all the pectins classified as high-methoxy pectins. The NMR (^1^H and ^13^C) analysis confirmed the presence of arabinans, galactans, galacturonans, and rhamnose, and the calculated DM and acetylation (DAc) values were consistent with the results obtained using conventional methods. These results provide insight into the influence of extraction methods on pectin properties, which is relevant to the pharmaceutical and food industries, and confirm the structural similarity between enzymatically extracted pectins and commercial pectin.

## 1. Introduction

Pectin substances are important components of plants [1]. The name “pectin” derives from the Greek word “pectos”, which means “to solidify, harden” and is a reflection of the gelling properties of these substances. Pectins are polysaccharides (heteropolysaccharides) that occur naturally in plants. They are found mainly in cell walls and the middle lamellae of intercellular spaces in plants [2,3]. Pectins serve multiple roles in plant tissues, such as contributing to structural integrity and controlling the water balance. Additionally, they fulfill another crucial function in plants by acting as an intercellular adhesive. Beyond their role in plants, pectic substances have medicinal benefits. Research has shown that they help prevent atherosclerosis by reducing blood cholesterol, binding harmful metals, and aiding in the regulation of intestinal movement. The amount of pectin in plant tissues varies depending on factors like the species, variety, and the anatomical part of the plant [1,4].

The main skeleton of pectin is composed of galacturonic acid residues (free and esterified with methanol), which are connected into long chains using α-1,4 glycosidic bonds, creating linear polymers. Depending on the degree of esterification, highly methylated pectins, in which the degree of esterification of carboxyl groups in galacturonic acid residues is more than 50%, may be distinguished from low-methylated pectins, in which the degree of esterification is below 50%. The degree of methylation (DM) refers to the proportion of carboxyl groups that have been esterified with methanol. The degree of acetylation (DAc) is defined as the percentage of galacturonosyl residues that have one acetyl group attached, assuming that it is the hydroxyl groups in galacturonosyl residues that are acetylated exclusively. Various oligosaccharide molecules may be attached to the main chain of pectin, the type of which is dependent on the plant from which they derive. They include a very complex group of polysaccharides, which are linked together covalently. The most important polysaccharides that form the structure of pectins are homogalacturonan, rhamnogalacturonan I, rhamnogalacturonan II, xylogalacturonan, arabinan, arabinogalactan I, and arabinogalactan II [5,6,7].

The diverse properties of pectins come from their structural variability at the molecular level, which can often be inferred via spectroscopic methods. These include both chemical analysis methods and advanced instrumental methods (NMR (Nuclear Magnetic Resonance), MS (mass spectrometry), IR (infrared spectroscopy), and Raman spectroscopy (FT-Raman spectroscopy as well). Among these methods, three were selected for use in the present study: NMR spectroscopy, FT-IR spectroscopy, and morphological analysis. The methods used provide information about the chemical structure of pectins through the identification of functional groups and the analysis of spectra. They can also be used to quantify or determine the extent of methylation and number of groups in a sample. Both types of spectroscopy (NMR and FT-IR) are used, for example, to control the reactions and to analyze the spectra of the products or their mixtures, as well as to determine the interactions occurring between the components in solutions. The main advantage of spectroscopic methods is their high sensitivity [1,2,3,4,5,6,7,8,9,10,11,12,13,14,15,16,17,18,19].

NMR spectrometry is a chemical technique that is utilized to gather insights about the structure and conformation of chemical compounds. This valuable method is used to characterize the composition and arrangement of polysaccharide units within pectin. It enables the quantification of functional groups in polymeric materials, as all the nuclei are equivalent and can produce signals of similar intensity, regardless of their chemical surroundings. To date, this spectroscopic approach has been employed to investigate the conformational chains of pectic macromolecules in both solid and gel forms. However, preparing pectin solutions for analysis poses challenges; although pectin dissolves readily in water, pectin solutions tend to be highly viscous, complicating the application of NMR solution techniques for analysis. In this study, we introduce a combination of analytical methods aimed at the quantitative determination and structural analysis of pectin in apples, utilizing 13C CP/MAS NMR spectra. In this work, we prepared 13C NMR spectra of apple pectins with different structures. Characteristic values, such as the degree of methylation (DM) and acetylation (DAc), were also obtained, based on the relative areas of C-6, COOCH_3_, and OCOCH_3_ resonance signals [20,21,22,23,24,25,26,27,28,29,30].

Apple pectins were chosen due to their specific degree of methoxylation, a characteristic demonstrated in prior research to be essential in regard to their ability to modulate viscosity and stabilize structures, which are properties valued in both food and pharmaceutical applications. Furthermore, the broad availability of apple pectins in a standardized form ensures experimental consistency and enhances the comparability of the results. This study aimed to examine the structure of apple pectin to support future investigations into the connection between its structure and biological activity. Pectins extracted from apples through the use of advanced enzymatic methods are distinguished as having a greater molecular mass and a more complex structure compared to other types of pectins, including those that are commercially available, which have been industrially processed. Enzymatic extraction is considered to be a more efficient and environmentally safe due to several factors: it generates wastewater that is generally easier to treat and dispose of compared to traditional methods like acid hydrolysis; the enzymes used are organic and biodegradable; and the process often utilizes low concentrations of enzymes, minimizing the potential environmental impact [31]. We aimed to determine the differences between apple pectins isolated using other methods and industrial apple pectin, which have not been previously examined using spectroscopic methods. Recently, FT-IR spectroscopy has been frequently used to determine the degree of methyl esterification (DM) of pectin [12], because this method does not require sample pretreatment. We intend to use the knowledge obtained for further research and chemical modifications to obtain new compounds that can be used in the food and pharmaceutical industries. FT-IR and NMR spectroscopy techniques were used for the analysis to outline the structure of the obtained pectin. This study provides theoretical guidance for further investigation of the chemical components of apple pectin.

## 2. Materials and Methods

### 2.1. Chemicals and Reagents

Deuterium oxide 99.9 atom % D was obtained from Merck (Darmstadt, Germany). All the reagents employed were of analytical grade quality.

Commercial, highly methylated apple pectin, isolated with sulfuric acid (P*_commercial_*), was obtained from Pektowin S.A., Jasło, Poland.

A set of calibration samples was prepared (Table 1). A series of mixtures of polygalacturonic acid and commercial apple pectin and a series of chemically demethyl esterified pectin samples were prepared to create pectin samples with different nanostructural features, including different DM and methyl esterification patterns.

### 2.2. Pectins

Pectins isolated from one batch of dried apple pomace were tested using enzyme preparations or hot water. The P*_cellulase_* symbol describes pectin extracted with endo-cellulase (endo-β-1,4-glucanase, EC 3.2.1.4, Sigma/Aldrich Chemical Co., Darmstadt, Germany, Cat. No. C9422) using doses of 50 U/g of pomace at pH 5.0 and 40 °C for 10 h, according to the method described by Wikiera et al. [10]. The symbols P*_arabinanase_* and P*_arabinanase+mannanase_* designate pectins released using endo-1,5-α-arabinanase (EC 3.2.1.99) and endo-1,4-β-mannanase (EC 3.2.1.78) (Megazyme, product code E-EARAB and E-BMANN). Both enzymes were used with a dose of 50 U/g of pomace at pH 5.0 and 40 °C for 10 h, according to the method described by Wikiera et al. [32]. The Pwater symbol describes pectin leached from apple pomace with water (20 mL/1 g of pomace at 85 °C for 10 h). Commercial apple pectin (P*_commercial_*) obtained from Pektowin (Jasło, Poland) was analyzed as a reference sample (Table 2) [33,34].

### 2.3. Instrumental Analysis

Morphological analysis was performed via microscopic analysis of the pectins, using an Eclipse Ti2 inverted microscope (Nikon Instruments Inc., Tokyo, Japan), with ×10 magnification. The dispersed powders acquired after isolation were transferred onto a microscope slide and analyzed using a series of photos to statistically determine shape and size descriptors. Images were taken in transmission and reflection illumination modes and were further analyzed using ImageJ software (1.54p) [31]. Microscopic images of freely separated pectin particles on glass substrates were analyzed. The analysis used specific illumination parameters to distinguish the particles from the background in grayscale images. A threshold was determined for each image based on pixel intensity histograms. A particle position mask was created and processed, using a watershed filter to separate touching particles. This mask was then analyzed to obtain the perimeter and roundness of the particles.

The qualitative properties of pectin were evaluated through the analysis of the attenuated total reflectance–Fourier transform infrared (ATR-FTIR) spectra. The pectin extracts were analyzed using the Nicolet iS50 FT-IR spectrometer system (Thermo Scientific Inc., Madison, WI, USA), equipped with a diamond ATR that allows for one-bounce reflectance. The FT-IR spectra were recorded in absorbance mode, covering a frequency range of 4000 to 400 cm^−1^, with a resolution of 4 cm^−1^, at ambient temperature.

The content of methyl esterification (DM) in pectin was found to correlate with the ratio of the band area at 1730 cm^−1^ to the total area of the bands at 1730 and 1630 cm^−1^ in the FT-IR spectrum [17]. To determine the DM content for various pectin samples, standard curves were created using pectin with known DM values. The peak areas at 1730 cm^−1^ and 1630 cm^−1^ were integrated and analyzed. A calibration curve was constructed based on pectin standards with an established % DM, using the equation $DM\%=\frac{A_{1730}}{A_{1730}+A_{1630}}$ [12]. The resulting linear correlation coefficient for this calibration curve was R^2^ = 0.9864. Data analysis was conducted using the Omnic software (https://www.thermofisher.com/order/catalog/product/INQSOF018, accessed on 31 March 2025).

The area corresponding to the esterified carboxyl peak was determined by calculating the region above the baseline within the range from 1844 ± 29.8 to 1682 ± 12.3 cm^−1^. In contrast, the area for non-esterified carboxyl peaks was measured above the baseline between 1682 ± 12.3 and 1532 ± 20.2 cm⁻^1^. The areas of both esterified and non-esterified carboxyl peaks were calculated using the area under the curve. The non-esterified carboxyl groups comprised both acidic and anionic species, which absorb at wavelengths of 1600 cm⁻^1^ and 1650–1550 cm⁻^1^, respectively [17]. In regard to this analysis, a standard pectin sample was utilized. To determine the ratio of the ester carboxyl peak area to the total carboxyl area of the pectin standard, the area under the curve for the esterified carboxyl region was divided by the combined areas of both esterified and non-esterified carboxyl peaks. Maintaining consistent sample preparation and processing conditions minimized the variability in the results caused by differences in particle morphology. The ^1^H NMR and ^13^C NMR spectra were collected using the Bruker 30 UltraShield Magnet system 300 MHz/54 mm (Bruker, Karlsruhe, Germany), at room temperature. Values were recorded at 300 MHz, in terms of the NMR analysis of pectin (35 mg), dissolved in 1 mL of high-quality D_2_O (99.96%). Moreover, the ^13^C CP/MAS spectra were recorded using a ^13^C and ^1^H RF field, with a frequency of 63 kHz and a spin rate of 15 kHz. In regard to the CP/MAS experiments, recycling delays of 3 s and 130 s were used. All the spectra were analyzed in reference to the carbonyl peak of glycine at 176.00 ppm. The spectra were apodized, with Lorentzian line broadenings of 10 Hz.

### 2.4. Chemical Shift Calculation

The ^13^C carbon chemical shifts were calculated using the CS ChemNMR Pro 6.0 and MestReNova 8.0 software.

The distribution of the ^13^C CP/MAS NMR spectra in the range of 180–170 ppm was performed using the peak fitting module in the Microcal Origin 6.0 software (Microcal Origin, Northampton, MA, USA). Another algorithm used in the present study supported the peak fitting procedure. The results of peak splitting were used to obtain the values of the degree of methylation (DM), amidation (DAm), and acetylation (DAc), based on the relative areas of the separated peaks.

The content of galacturonic acid (GalA) in pectins was determined photometrically with *m-*hydroxybiphenyl (520 nm), which corresponded to the total content of uronic carboxyl groups. The DAc values for strongly acetylated (>1) samples were obtained with solid-state NMR spectroscopy, using OCOCH_3_ resonance as a marker of acetyl groups. For strongly acetylated samples (DAc > 1), the DAc value was determined using solid-state NMR spectroscopy. The degree of amidation (DAm) of amidated pectins was calculated based on elemental analysis. The DM, Dam, and DAc values were expressed as the relative content of methoxy (%), amide (%), and acetyl (mol/mol) groups.

All the experiments were performed in regard to at least three independent replicates. In regard to the morphological analyses, FT-IR and NMR spectroscopy were performed on pectin samples subjected to the purification procedure, which ensures that the obtained data characterize the structure of pure pectins [10,31,34,35].

## 3. Results and Discussion

### 3.1. Morphology Analysis

**Figure 1:**
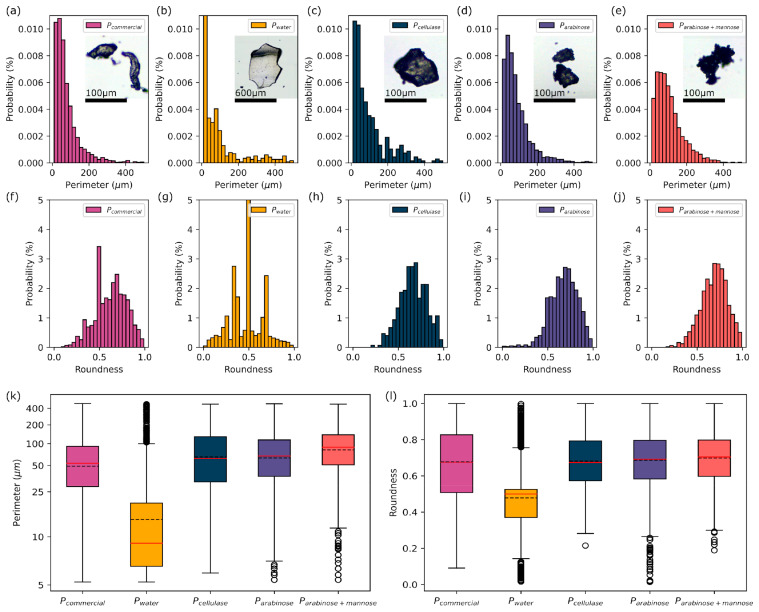
Statistical description of pectin morphology for perimeter (**a**–**e**), and roundness (**f**–**j**) for subsequent pectins: (**a**,**f**) P*_commercial_*; (**b**,**g**) P*_water_*; (**c**,**h**) P*_cellulase_*; (**d**,**i**) P*_arabinanase_*; and (**e**,**j**) P*_arabinanase+mannanase_*. The inset contains a photo of a representative pectin pellet. Boxplot showing (**k**) the perimeter, based on a logarithmic scale, and (**l**) the roundness, based on a normal scale, for consecutive pectin samples. The dashed lines indicate the mean and the solid red lines indicate the median.

The studied pectins are characterized by morphological differences, affecting both the size and shape of the precipitates. The size distribution in most of the investigated samples had a log-normal distribution, which is characteristic of the statistical distribution of particles, similar to [36]. For ease of interpretation, the perimeter results in Figure 1k are presented based on a logarithmic scale, with the values based on a normal scale. The perimeter of P*_water_* pellet particles contains a significant number of outliers. There are juxtaposed smaller particles with a log-normal size distribution and a significant number of larger structures that cause the overall distribution to be non-log-normal. Based on all the tested samples, a significant difference was observed in regard to P*_water_*, according to which the coexistence of three particle populations was visible. A moderate particle size, with a perimeter of <200 μm, forming a log-normal distribution, can be observed, along with structures with a peak perimeter at about 400 μm, visually presenting a structural difference (Figure 1a–e). Despite the existence of a significant number of small structures in terms of the P*_arabinose_* and P*_arabinose+mannose_* pectins that are outliers, as seen in Figure 1k, the log-normal distribution for these materials has a low skewness value that decreases in regard to the series, P*_cellulase_,* P*_arabinose_,* and P*_arabinose+mannose_*, with a concomitant increase in the median value. Smaller sized pectin particles are associated with more efficient pectin release from cell walls [37,38]. Some large, single structures of P*_water_* were detected, but did not impact the statistical description. Despite the chemical similarity, significant differences in shape were observed. The pectin with the most compact and spherical structure was P*_cellulase_*, while P*_arabinanase_* and P*_arabinanase+mannanase_* have a non-uniform surface (Figure 1l), significantly increasing the surface area, while maintaining moderate–high roundness, defined as $4\cdot A/(\pi \cdot d_{M}^{2})$, where A is the particle area and d_M_ is the major axis length when the particle is approximated by an ellipse. The rough external structure may be a direct result of the enzymatic extraction and has a positive effect on the increased total particle surface area, which might facilitate cholesterol binding and antioxidant activity [32,37,38], as well as the emulsifying activity [33]. P*_commercial_* pectins have an elongated shape, resulting in a wide distribution of roundness values. The shape of P*_water_* also shows significant differences; the pectin is in the form of thin flakes, with low light scattering and a large size (see inset to Figure 1). Among the materials tested, P*_water_* should be identified as the sample with the greatest diversity in terms of both roundness and the perimeter. It was noted that ultrasound isolation makes it possible to obtain pectin with expanded, irregular surface “grains” [39].

### 3.2. Characterization of Pectin via FT-IR Spectroscopy

**Figure 2:**
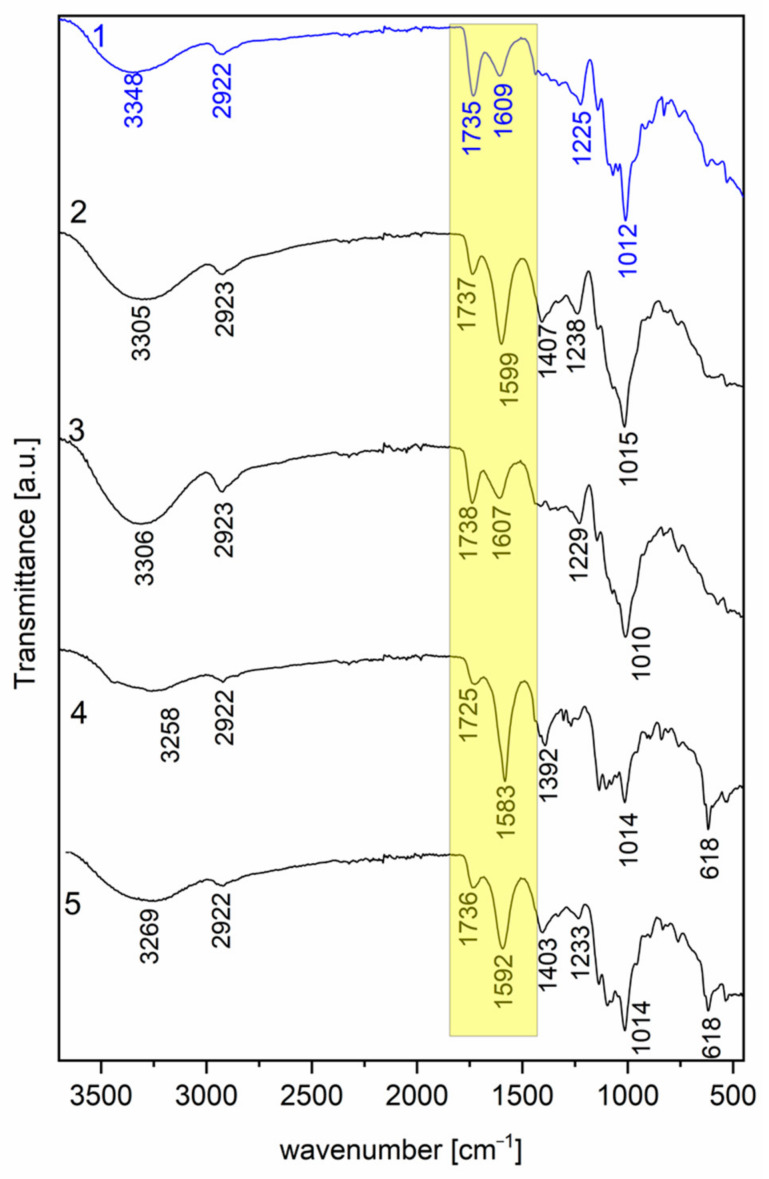
FT-IR spectra (left) of commercial apple pectin (**1**) and apple pectin extracted with cellulase (**2**), hot water (**3**), arabinanase and mannanase (**4**), and arabinanase (**5**), and the images from the confocal microscope before the measurement of FT-IR spectra of the discussed pectins (**1**–**5**) (right); yellow column: bands correspond to the stretching vibration of (C=O) in the methyl esterified carboxyl group (COO-R) and the C=O stretching vibration in (COO^−^).

The FT-IR spectra of P*_commercial_* and the pectins extracted from apple pomace with hot water and enzymes are presented in Figure 2. The primary purpose of the described spectroscopic study is to identify potential alterations in the structures of pectin following its extraction from apple pomace.

Just like in all carbohydrates, the prominent feature of the valence vibrations of the hydroxyl groups within the pyranose or furanose ring, represented by the ν(O-H) band, is a distinctive trait of pectin. The primary peaks in the spectral range of 3258–3306 cm⁻^1^ (3348 cm⁻^1^ in the case of **1**) for samples **2**–**5** are a result of hydroxyl group stretching, with a noticeable shift towards lower frequencies compared to sample **1**. Overall, the shift in the O-H stretching vibration bands for pectins **2**–**5** towards lower frequencies is often associated with the formation of hydrogen bonds and changes in the chemical environment of the hydroxyl groups. When the hydroxyl group (O-H) forms hydrogen bonds with other groups, the energy required to stretch the O-H bond decreases, causing the absorption band to shift towards lower frequencies. A decrease in the number of water molecules associated with pectins can also cause a shift in the O-H stretching vibration bands. Water influences hydrogen bonds, and its presence or absence can change the O-H stretching frequencies. In conclusion, samples **2**–**5** can be less hydrated compared to sample 1. The stretching vibrations of the C-H groups in regard to CH_2_ generate medium-intensity bands at around 2922 cm⁻^1^ for samples **1**, **4**, and **5** and 2923 cm⁻^1^ for samples **2** and **3**. It is worth noting that the frequency range of 3000–2800 cm⁻^1^ is commonly associated with ν(C-H) stretching vibrations [11,12]. Assigning these vibrations to specific groups, such as methyl and methylene groups or the pyranose ring of pectin, can be challenging due to the overlap with broader ν(O-H) stretching vibrations [13,14]. In general, the presence of hydrogen bonding interactions leads to a downward shift in the stretching frequencies of ν(C=O) and ν(O-H). This phenomenon indicates that pectin structures are stabilized by intermolecular hydrogen bonds [15]. Following this, medium-intensity bands are observed at approximately 1735 cm⁻^1^ and medium bands at 1609 cm⁻^1^ (in the case of **1**). These bands correspond to the stretching vibration of ester (C=O) in the methyl esterified carboxyl group (COO-R) and the C=O stretching vibration in regard to the ionic carboxyl groups (COO^−^), respectively (yellow column in the spectra, Figure 2). It is worth noting that the intensity of these two bands is inverted when compared to the FT-IR spectra of samples **2**–**5**: a medium band around 1725–1738 cm⁻^1^ and a strong one ranging from 1583 to 1607 cm⁻^1^ are observed in the FT-IR spectra of samples **2**–**5**, in contrast to sample **1**. The weak band near 1225 cm⁻^1^ is attributed to side-chain vibrations. Structural characteristics related to specific conformations around the glycosidic bonds of pectin can be observed in the spectral range of 1100–900 cm⁻^1^. Additionally, there is a band corresponding to the ring vibration coupled with δ (C-OH) bending vibrations, which are notably weak in this range [13,16]. In the research presented, the prominent band with a strong intensity at around 1012 cm⁻^1^ (in **1**) in the FT-IR spectra of pectins is associated with C-O stretching vibrations. The remaining medium-intensity bands below 900 cm⁻^1^ in the “fingerprint” region are primarily attributed to vibrations of the C-O-C bridges, which are typical for polysaccharides [17,18].

In conclusion, the best spectroscopic matching is found in regard to samples **3** and **1**, indicating a very similar structure for these apple pectins. This observation is supported by the results of the FT-IR analysis (Table 3).

Comparing the DM obtained using the FT-IR method for individual pectins, we can see that pectin (**2**) has the highest DM of 58% and pectin (**4**) has the lowest DM of 30%, which is caused by the production process. Pectins **1**–**5** can be classified as high-methoxy pectins.

The DM content for pectins **1**–**5**, determined using the FT-IR method, is shown in the table below (Table 4).

### 3.3. Characterization of Pectin via ^1^H NMR Spectroscopy

NMR spectroscopy, although demanding, is a valuable tool in pectin analysis. Despite its lower resolution and sensitivity compared to solution NMR spectroscopy, this technique provides important information on the structure of pectins. Analysis of the OCH_3_ and CH_3_ resonances as ester and acetyl markers and peak matching in regard to the C-6 spectral region enable the estimation of the proportions of the different C-6 carboxyl groups and O-acetyl groups in pectin.

**Figure 3:**
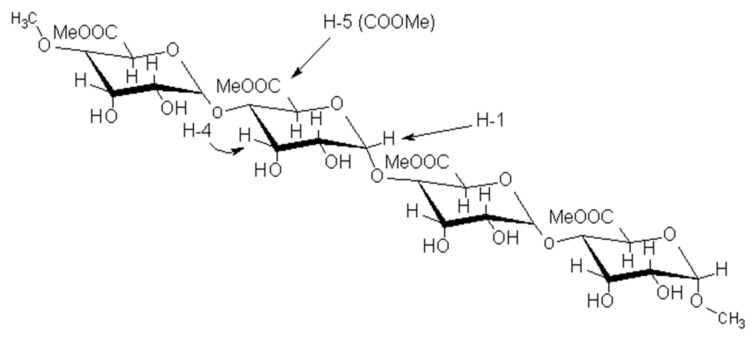
Structure of a fragment of apple pectin.

To determine the degree of methylation (DM), the H-5 integrals (Figure 3) adjacent to the ester (*I*_COOMe_) were compared with the sum of the H-5 integrals adjacent to the ester (*I*_COOMe_) and the H-5 integrals adjacent to the carboxylate (*I*_COO−_).

Due to the overlap or proximity of the signals for H-1 and H-5 (COOMe) integrals, only the described combinations for H-1 and H-5 (COOMe) integrals are possible [19,20].

The NMR spectroscopy analysis depicted in Figure 2 represents the ^1^H spectra of commercial apple pectin (**1**) and apple pectin extracted with cellulase (**2**), hot water (**3**), arabinanase and mannanase (**4**), and arabinanase (**5**). The presented chemical shifts are based on a comparison with the already published spectral data on the structural characteristics of pectins [21,22,23,24,25,26]. The signals were detected in regard to pectin-type spectra, more precisely related to arabinose, galactose, galacturonate, and rhamnose (Table 5).

**Figure 4:**
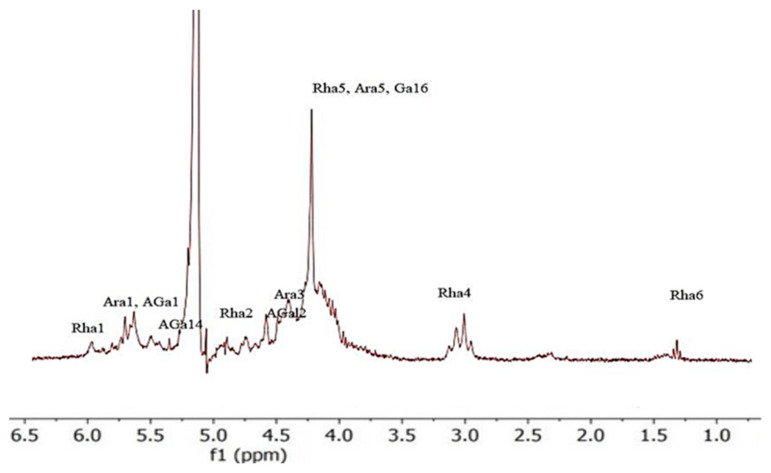
^1^H NMR spectra of apple pectins (**5**).

**Figure 5:**
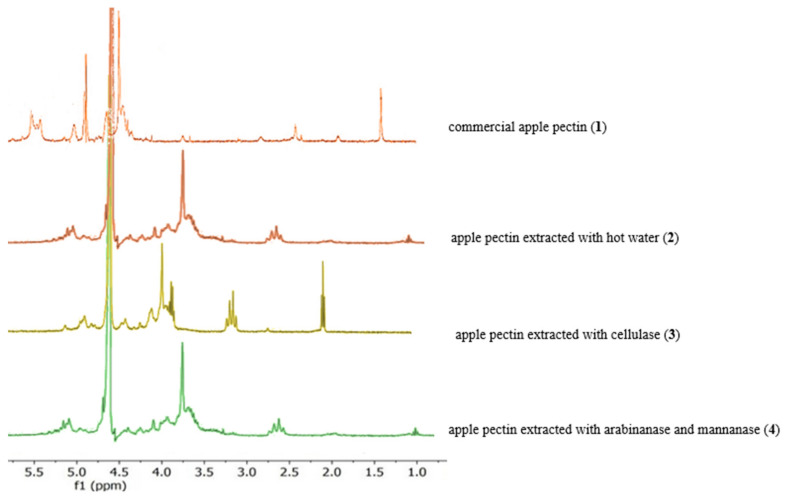
^1^H NMR spectra of apple pectins (**1**–**4**).

The ^1^H spectrum showed a high level of similarity to the presented results [12,20]. The area in regard to the anomeric signals, as shown in Figure 4 and Figure 5, contained at least four signals at 4.81–5.23 and 4.96 ppm for samples (**1**–**3**). These were assigned as follows: α-(**1**→**2**) to rhamnopyranosil, α-(**1**→**5**) to arabinofuranosil, β-(**1**→**4**) to galactopyranosyl and acid and α-(**1**→**4**) linkages to galactopyranosyl. We can identify three triplets centered at 1.27 and 1.38 ppm for the sample (**1**–**3**) fractions, which are assigned to the six H-rhamnose residues. Units that are only observed in samples (**1**→**2**) for galacturonic acid correspond to a signal at ~1.24 ppm. In contrast, the signal at ~1.38 ppm corresponds to the rhamnose units linked (**2**→**1**) to the *O*-4 branch of galacturonic acid.

### 3.4. Characterization of Pectin via ^13^C NMR Spectroscopy

Weak resonance at ~55 ppm was present in the spectra of pectins **1**–**3**, indicating a much more marked methyl ester group in these samples. Resonances at 175–167 ppm are assigned to the C-6 carbons of the galacturon units, while carbons belonging to OCOCH_3_ acetyl groups are also found in this region.

They are formed from carbon glycoatoms with a side bond, C-1 and C-4, assigned to peaks of ~100 ppm and ~80 ppm, respectively. The peaks observed between 67 and 72 ppm originate from the other carbons in the pyranose ring. Conversely, acetylation significantly alters the resonance signals of these pyranose ring carbons (Table 6 and Table 7) [24,25,26,27,28]

A weak resonance around ~55 ppm was detected in the spectra of pectins **1**–**3**, suggesting a significantly higher presence of methyl ester groups in these samples. The resonances observed between 175 and 167 ppm are attributed to the C-6 carbons of the galacturonic units, and this region also contains signals from the OCOCH_3_ acetyl groups. These are generated from carbon glycoatoms with side bonds, specifically C-1 and C-4, which correspond to peaks at approximately ~100 ppm and ~80 ppm, respectively. The peaks ranging from 67 to 72 ppm originate from additional carbons in the pyranose ring. In contrast, acetylation causes notable alterations in the resonance signals of pyranose ring carbons [24,26]. The upfield shift of C-2, 3, and 5 resonances observed in pectins **1**–**3** could be due to both methylation, indicated by the shift in the C-5 resonance, and acetylation, which reflects the dominance of diacetylated units. The C-1 carbon is involved in forming the α(**1**→**4**) glycosidic bond in the pectin backbone, making it more flexible compared to the other carbons in the pyranose ring [27,28,29].

**Figure 6:**
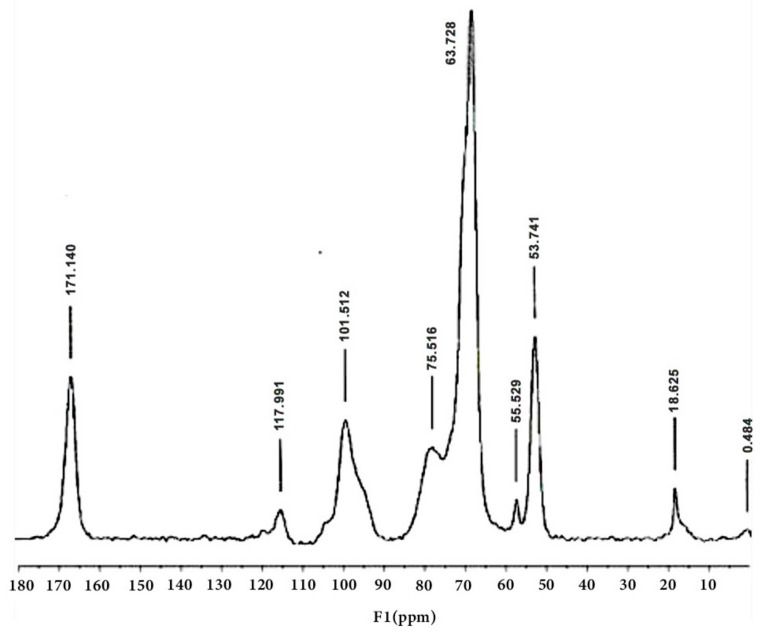
^13^C CP/MAS solid-state NMR spectrum of commercial pectin (**1**).

**Figure 7:**
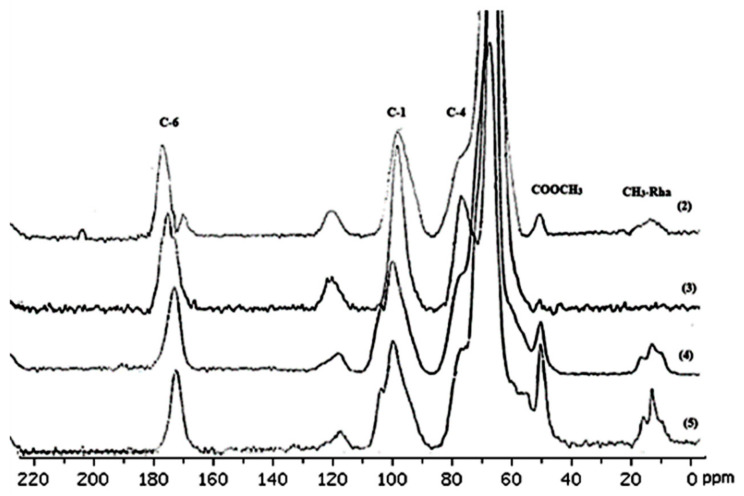
^13^C CP/MAS solid-state NMR spectra of pectins (**2**–**5**).

In the ^13^C NMR spectrum (Table 7), the area of 190–160 ppm belongs to the carboxyl carbon atoms (C-6) of galacturonic units in the form of carboxylic acid COOH, carboxylate anion COO–, and ester COOCH_3_ [30]. The resonance signals of the carboxyl carbon atoms in the OCOCH_3_ acetyl groups present in apple pectins cannot be separated due to the overlapping of the intense C-6 (COOCH_3_) signal (Figure 6 and Figure 7) [30,31,32,35,36,37,38].

The resonances of the C-6 carbon (COOCH_3_) are affected by the positioning of methyl ester groups within the pectin structure [31]. However, challenges arise when utilizing ^13^C CP/MAS NMR spectroscopy to investigate pectin structures, particularly in regard to amidated and highly acetylated variants. The resonance signals for the carboxylic carbon in the pectin spectra tend to be too broad, making it difficult to distinguish them from one another. Despite this, it is possible to mathematically isolate these signals from the overall C-6 resonance (COOCH_3_) through the use of peak separation techniques. Peak chance analysis, commonly employed in chromatography and infrared spectroscopy for biopolymers, facilitates the resolution of components within complex spectral regions, enabling both qualitative and quantitative assessment of the sample. Important parameters, such as the degree of methylation (DM) and acetylation (DAc), were derived from the relative areas of the C-6, COOCH_3_, and OCOCH_3_ resonance signals. It has been demonstrated that the C-6 region (180–160 ppm) of the spectra is particularly valuable for the structural characterization of pectin. These findings are supported by the studies conducted by Synitrya [26,31].

The quantities of methyl ester and acetyl groups were determined by analyzing the areas of the respective Lorentzian, Gaussian, and Voigt components (see Table 8). For apple pectin, the mixed Lorentz–Gauss (Voigt) function provided a significantly more accurate full-state NMR solution compared to the individual Gauss or Lorentz functions. The measured values of DM and DAc closely aligned with those obtained through the use of conventional methods, including the NMR technique, which relies on the relative areas of COOCH_3_ and OCOCH_3_ resonance signals [30,31,32,33,35,36,37,38,39].

## 4. Summary

These studies provide valuable insights into the influence of extraction methods on pectin properties, focusing on morphological and structural analysis. The results demonstrate that extraction methods significantly affect pectin morphology, manifesting as differences in particle size and shape. Enzymatic extraction, particularly using arabinase and arabinase with mannanase, yielded pectins with an uneven surface, potentially increasing their specific surface area. This characteristic may be advantageous in industrial applications where interactions with other substances, such as cholesterol binding, antioxidant, or emulsifying activity, are crucial. Commercial pectin and water-extracted pectin exhibited distinct morphological features compared to enzymatically extracted pectins. The FT-IR analysis revealed changes in the pectin structure depending on the extraction method, suggesting that modifications occurred in regard to hydrogen bonding and hydration. The degree of methoxylation (DM) varied with the extraction method, impacting the functional properties of pectins. The ^1^H and ^13^C NMR analysis confirmed the structural differences among the pectins obtained using various methods and provided data on the degree of acetylation. New pectins, especially those obtained enzymatically, displayed unique morphological and structural properties that could be beneficial in industrial applications. The increased specific surface area of enzymatic pectins may enhance their ability to bind bioactive substances, which is relevant in the pharmaceutical and food industries. Variations in the DM and acetylation influence the rheological properties of pectins and their gel-forming capabilities, which are crucial in the food industry. Structural differences among the pectins obtained using different extraction methods enable their potential use in diverse applications, which means that pectins can be tailored to address specific needs.

The FT-IR and NMR spectroscopic methods proved to be fundamental and highly informative tools for pectin analysis. The FT-IR methods for determining the degree of methoxylation are applicable in various industries, such as food, pharmaceuticals, and cosmetics. The DM values obtained using FT-IR spectroscopy were in good agreement with those calculated as a result of standard techniques. However, the DM results from the FT-IR and NMR spectroscopy differed significantly. This manuscript demonstrates that hydrolysis and sample purification are essential for reliable DM and DAc determinations using NMR analysis. Spectroscopic methods without prior hydrolysis are highly effective for studying the structure of polymers like pectin. The correlation between the spectral features and functional properties of pectins was established, with changes in the degree of methoxylation (DM) and acetylation being linked to significant modifications in their rheological properties, including gel-forming capabilities. The enzymatically extracted pectins exhibited an increased specific surface area, which enhances its ability to bind bioactive substances, with potential benefits for pharmaceutical and food industries. The structural differences observed in regard to these pectins suggest that they could be tailored for specific industrial applications, such as in regard to their cholesterol binding, antioxidant activity, and emulsifying properties.

The morphological characterization of materials is crucial for industrialists and scientists. Polymers, including apple pectin, often exhibit specially designed morphologies and microstructures that have specific properties. Therefore, understanding the structure–property relationships of materials is essential for predicting pectin behavior. These studies utilized spectrometric chemical analysis methods (^1^H NMR, ^13^C NMR, ^13^C CP/MS NMR, and FT-IR spectroscopy), incorporating some features of apple pomace components. The primary objective of spectroscopic studies is to detect changes in pectin following its extraction from apple pomace. A key feature of pectin is the valence vibration of hydroxyl groups in the pyranose ring, characterized by the ν(O-H) band. The FT-IR spectra revealed that the stretching peaks of the hydroxyl groups for samples **2**–**5** were shifted to lower frequencies compared to the Pcommercial sample, indicating hydrogen bond interactions that stabilize pectin structures due to intermolecular bonds. This study showed that the quality of the pectins in samples **2**–**5** differed from that of commercial pectin (**1**), with sample **3** exhibiting the greatest structural similarity, confirmed by the FT-IR analysis. The DM results, based on NMR spectra, are obtainable after the hydrolysis process has taken place. The DM results obtained from these spectra justify the use of the aforementioned hydrolysis process to achieve consistent research results, as demonstrated in this manuscript. While FT-IR and NMR spectroscopy are both fundamental tools for pectin analysis, discrepancies in the DM values between these methods were observed. These differences likely arise due to variations in sample preparation, hydrogen bonding effects, and the specific molecular interactions captured by each technique. FT-IR analysis provides rapid DM estimation based on characteristic absorption bands, yet its accuracy may be influenced by overlapping signals or hydration state variations. In contrast, NMR analysis offers a more precise DM determination, but requires hydrolysis to take place, which may introduce structural modifications. The need for hydrolysis in regard to NMR analysis highlights a crucial methodological consideration, as improper sample preparation can lead to inconsistencies in DM and DAc values. These findings underscore the importance of selecting the appropriate spectroscopic method, depending on the specific application and desired level of accuracy.

However, further studies are necessary to precisely determine the effect of extraction methods on the molecular weight and functional properties of pectins, establish correlations between the spectral features and functional properties, conduct comparative studies between spectroscopic methods to analyze discrepancies in the results, and investigate the kinetics of enzymatic reactions and the impact of extraction parameters on pectin properties. In summary, these studies provide valuable information on the effect of extraction methods on pectin properties. New pectins, particularly those obtained enzymatically, exhibit promising properties for various industrial applications. However, further research is needed to fully understand their potential.

## Figures and Tables

**Table 1:** Basic chemical composition of commercial pectin (P*_commercial_*) [10].

M_w_ (kDa)	GalA (%)	DM (%)	NS * (%)	Protein (%)	Reactive with F–C Reagent (%)
378 ± 45	80.9 ± 2.4	56.9 ± 2.1	14.3 ± 0.7	0.78 ± 0.12	0.49 ± 0.08

NS *—total neutral sugars.

**Table 2:** Extraction yield and molecular weight (M_w_) of tested pectins [32,34].

Pectin	Yield (% Wet Basis)	M_w_ (kDa)	Source
P*_commercial_*	-	378 ± 45	[10]
P*_cellulase_*	15.20	589 ± 66	[10,35]
P*_arabinanase_*	16.34	452 ± 8	[35]
P*_arabinanase+mannanase_*	13.51	341 ± 6	[31]
P*_acid_*	13.33	312 ± 5	[31]

**Table 3:** Integration area and peak height used to calculate DM in FT-IR analysis.

FT-IR Band (cm⁻^1^)	Assignment	Integration Area/Peak Height	Application in DM Calculation	References
~1740–1750	Carboxyl esters (C=O)	Integration area or peak height	Proportional to the content of the methoxyl groups	[12,14,18]
~1630–1600	Free carboxyl groups (COO⁻)	Integration area or peak height	Proportional to the content of free galacturonic acid groups	[12,14,18]
Ratio of 1740/1630	Ratio of peak height or area integral	Ratio of esterified carboxyl groups compared to free carboxyl groups	The ratio of the peak area or height of these two bands is used to calculate the degree of methoxylation (DM)	[12,14]

**Table 4:** The degree of methoxyl groups present in pectins.

PECTIN		%DM *
P*_commercial_*	(**1**)	57% ± 0.5%
P*_cellulase_*	(**2**)	58% ± 0.3%
P*_water_*	(**3**)	51% ± 0.5%
P*_arabinose+mannose_*	(**4**)	30% ± 0.34%
P*_arabinose_*	(**5**)	36% ± 0.4%

* The findings are presented along with the corresponding standard errors of the mean (± SEMs) calculated from the average of the quadruplicate values. The degree of methyl esterification (DM) refers to the mole percentage of carboxyl groups that have been esterified with a methyl group [12]. The pharmacological effects of pectin are influenced by the extent of DM formation.

**Table 5:** Comparing ^1^H NMR analysis of apple pectin with data in the literature, with references to the scientific articles [21,22].

Feature	Apple Pectins	Apple Pectin from Literature 1 [21]	Apple Pectin from Literature 2 [22]
Galacturonic Residue Distribution	Random or block	Block	Random or block
Presence of Acetyl Groups	Yes	Yes	Yes
Presence of Other Sugars	Rhamnose, arabinose, galactose	Rhamnose, arabinose, galactose	Rhamnose, arabinose, galactose, xylose
Region 3.0–4.0 ppm (Protons in the Galacturonic Ring)	Broad signals	Broad signals	Broad signals
Region 4.8–5.2 ppm (Anomeric Protons)	Multiple signals	Multiple signals	Multiple signals
Region 3.7–3.8 ppm (Methoxy Protons)	Singlet	Singlet	Singlet
Region 2.0–2.2 ppm (Acetyl Protons)	Singlet	Singlet	Singlet
Region 1.2–1.3 ppm (Rhamnose Protons)	Doublet	Doublet	Doublet
Other Significant Signals	-	-	-

**Table 6:** ^13^C NMR chemical shifts [12,25,26,27].

Chemical Shift Assignment (ppm)	Structure/Functional Group	Justification from the Literature	References
170–175	Carboxyl groups (C=O) in galacturonic acid	Characteristic shift of carboxyl groups in uronic acids.	[12,25,26]
100–105	Anomeric carbons (C-1)	Characteristic shift of anomeric carbons in polysaccharides.	[12,26]
70–85	Galacturonic ring carbons (C-2, C-3, C-4, C-5)	Shifts in this range correspond to the carbons in the sugar ring.	[25,27]
52–55	Methoxy groups (OCH_3_)	Characteristic shift of methoxy groups.	[12,26]
20–22	Acetyl groups (COCH_3_)	Characteristic shift of acetyl groups.	[25,26]
17–18	Rhamnose carbons (C-6)	Characteristic shift of methyl groups in rhamnose.	[12]

**Table 7:** Assignments for the peaks in the ^13^C NMR spectra.

Polymer	Carbon	Shift (ppm)		Sample	C-1	C-2,C-3,C-5	COOCH_3_	OCOCH_3_
*Galacturonan*	C-6	173.31		1	98.9	65.7	54.2	21.9
*Galacturonan*	C-6	171.27		2	101.6	66.1	54.4	21.2
*Arabinan*	C-1	105.6	^13^C NMR	3	101.9	66.1	54.6	19.6
*Galacturonan*	C-1	100.56	chemical shifts	4	99.6	65.5	54.2	21.0
*Galactan*	C-4	78.84	for the	5	101.0	66.1	54.5	19.9
*Galacturonan*	C-3	71.23	samples of					
*Galacturonan*	C-2	69.12	pectins.					
*Arabinan*	C-5	68.92						
*Galacturonan*	OCH_3_	53.54						
*Rhamnose*	CH_3_	17.22						

The observed chemical shifts are consistent with those reported by Tamaki et al. [31].

**Table 8:** Obtained degree of methylation (DM) and acetylation (DAc) of ^13^C CP/MAS NMR spectra.

Area, 10^6^					
Sample	A^1^_COOCH3_ 34	A^2^_COOCH3_ (C-6)	A_OCOCH3_	DM (%)	DAc (%)
**1**	25.80	45.90	19.92	58.38	0.43
**2**	37.84	58.23	78.79	64.98	1.35
**3**	17.34	52.54	77.79	33.00	1.48
**4**	37.43	51.33	78.79	72.93	1.53
**5**	33.88	51.44	77.79	65.87	1.51

DM(%) = A^1^_COOCH3_/A^2^_COOCH3_ × 100; DAc = A_OCOCH3_/A^2^_COOCH3_.

## Data Availability

The data will be made available on request.
